# Baseline PSMA PET/CT Biomarkers for Patient Selection in [^177^Lu]Lu-PSMA Radioligand Therapy: Clinical Promise, Methodological Heterogeneity and Future Standardization

**DOI:** 10.3390/ph19071084

**Published:** 2026-07-14

**Authors:** Grytė Galnaitienė, Matas Šimkus, Ieva Balčiūnaitė, Kornelija Lušaitė, Fang Wen, Marco Hoffmann, Matthias Saar, Felix M. Mottaghy, Donatas Vajauskas, Susanne Lütje

**Affiliations:** 1 Department of Radiology, Medical Academy, Lithuanian University of Health Sciences, LT-44307 Kaunas, Lithuania; gryte.galnaitiene@lsmu.lt (G.G.); kornelija.lusaite@lsmu.lt (K.L.);; 2 Faculty of Medicine, Medical Academy, Lithuanian University of Health Sciences, LT-44307 Kaunas, Lithuania; 3 Department of Nuclear Medicine, University Hospital RWTH Aachen, 52074 Aachen, Germany; 4 Department Urology and Pediatric Urology, University Hospital RWTH Aachen, 52074 Aachen, Germany; 5 Center for Integrated Oncology (CIO), University Hospital RWTH Aachen, 52074 Aachen, Germany; 6 Department of Radiology and Nuclear Medicine, Maastricht University Medical Center, 6202 Maastricht, The Netherlands

**Keywords:** PSMA PET/CT, biomarkers, PSMA therapy, patient selection

## Abstract

PSMA PET/CT imaging biomarkers are increasingly investigated as predictors of response and survival outcomes following [^177^Lu]Lu-PSMA radioligand therapy in prostate cancer. This review summarizes the current evidence and clinical relevance of these biomarkers. As [^177^Lu]Lu-PSMA radioligand therapy is increasingly being introduced into earlier treatment lines of prostate cancer management, the identification of reliable imaging biomarkers for response prediction and outcome assessment is becoming increasingly important. Quantitative PSMA PET/CT parameters such as whole-body (WB) mean standardized uptake value (SUV_mean_), WB PSMA-positive tumor volume, and tumor load, as well as qualitative features including disease distribution and phenotype (e.g., visceral metastases and FDG-avid, PSMA-negative discordant lesions), have been associated with treatment response, progression-free survival, and overall survival. However, the clinical utility of these imaging biomarkers is limited by heterogeneous study designs, small cohorts, and a lack of standardization in image acquisition, quantification, and cutoff definitions. This review critically appraises available data on baseline PSMA PET/CT-based prognostic and potentially predictive biomarkers for RLT and identifies the methodological barriers that must be addressed before these biomarkers can be implemented in clinical practice.

## 1. Introduction

Prostate-specific membrane antigen (PSMA)-targeted radioligand therapy (RLT) with [^177^Lu]Lu-labeled small-molecule ligands has changed the treatment of metastatic castration-resistant prostate cancer (mCRPC). The VISION trial showed that [^177^Lu]Lu-PSMA-617 improves radiographic progression-free (rPFS) and overall survival (OS) in PSMA-positive mCRPC patients who progressed after androgen receptor pathway inhibitors and taxane chemotherapy [1]. The TheraP trial demonstrated comparable OS with fewer grade 3–4 adverse events versus cabazitaxel while using more strict PSMA positron emission tomography/computed tomography (PET/CT)-based eligibility criteria [2,3]. As RLT moves into routine use and earlier treatment lines [4], optimizing patient selection has become a clinical priority.

PSMA PET/CT provides both the eligibility assessment and several imaging parameters, such as uptake intensity, tumor burden and disease distribution patterns, that have been associated with RLT outcomes across multiple studies. Whether these parameters can be used as patient selection tools depends on the quality and comparability of the underlying evidence.

**Figure 1:**
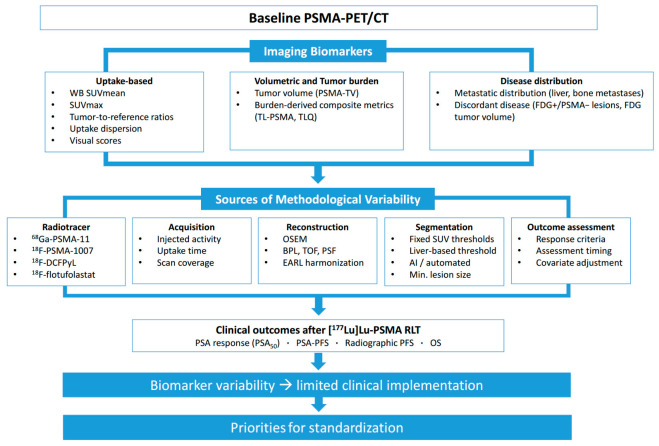
Overview of baseline PSMA PET/CT imaging biomarkers, sources of methodological variability, and their impact on clinical implementation of [^177^Lu]Lu-PSMA radioligand therapy.

To date, no quantitative PSMA PET biomarker has been validated for clinical use. Patient selection for RLT still relies on visual assessment as defined in the VISION trial [1]. Studies differ in imaging protocols, quantification methods, and study design, which limits the direct comparability of published values across settings. The aim of this narrative review is to summarize the existing evidence on baseline PSMA PET/CT biomarkers for patient selection, analyze the sources and consequences of methodological variability across studies, and propose priorities for standardization in future research. An overview of the review structure is presented in Figure 1.

## 2. Baseline PSMA PET/CT Biomarkers for Pre-Treatment Patient Selection

Baseline PSMA PET/CT biomarkers proposed for predicting RLT outcomes can be broadly grouped into three categories: uptake-based; volumetric and tumor burden; and disease distribution metrics.

### 2.1. Uptake-Based Biomarkers

The therapeutic effect of RLT depends on sufficient PSMA expression in tumor lesions, which can be estimated through the intensity of PSMA uptake on PET/CT. The rationale is straightforward: higher PSMA expression should increase tumor-absorbed [^177^Lu]Lu-PSMA doses and improve targeting, resulting in better outcomes [5]. Several studies have therefore investigated whether uptake metrics can predict RLT outcomes, but the results are not consistent.

**Quantitative SUV measures**

Whole-body (WB) tumor mean standardized uptake value (SUV_mean_), defined as the average uptake across all segmented tumor lesions, is the most extensively studied uptake-based biomarker for predicting RLT outcomes. By integrating PSMA uptake intensity across the entire tumor burden, WB SUV_mean_ reflects both overall PSMA expression and intrapatient heterogeneity.

In VISION trial secondary analysis, WB SUV_mean_ was the best predictor of RLT outcomes that outperformed maximum SUV (SUV_max_), tumor volume (TV), and tumor load (TL) [6]. Higher baseline WB SUV_mean_ was associated with better radiographic progression-free survival (rPFS), overall survival (OS) and higher odds of overall response rate (ORR) and prostate-specific antigen (PSA) response. Importantly, the association appeared approximately linear, and no clear cutoff value could be identified within the already visually selected PSMA-positive population.

Similar findings have been observed across multiple cohorts with various cutoffs and endpoints [7,8,9,10], supporting the association between higher SUV_mean_ values and better RLT outcomes. A recent meta-analysis confirmed the prognostic value of SUV_mean_ for OS (pooled HR 0.93), PFS (HR 0.88), and PSA response (OR 1.40), whereas SUV_max_ and SUV_peak_ showed no clinically meaningful associations [11]. Notably, in an independent multicenter cohort, WB SUV_mean_ remained the strongest independent prognosticator after adjustment for clinical variables in multivariable analysis, and outperformed other PET/CT visual scores and composite metrics [10]. In the TheraP trial, patients with SUV_mean_ ≥ 10 had significantly higher PSA response rates with RLT than with cabazitaxel, suggesting predictive value for treatment selection [12]. For survival, however, the signal was prognostic rather than predictive: in the mature OS analysis, higher SUV_mean_ was associated with longer survival in both treatment arms, regardless of received treatment [3].

Other uptake-related parameters also showed predictive value. At the patient level, Ozkan et al. found that baseline SUV_max_, SUV_peak_, SUV_mean_, and tumor-to-salivary-gland ratios predicted PSA response and disease control but could not distinguish responders from non-responders by RECIP criteria [13]. At the lesion level, Groener et al. showed that baseline SUV_max_, SUV_mean_, and the tumor-to-liver ratio were moderately correlated with molecular lesion response, although clinically used liver-based uptake thresholds had limited value in identifying nonresponding lesions—even lesions with uptake below the liver responded in 45% of cases [14].

Uptake dispersion may carry additional information. Kind et al. found that the interquartile range (IQR) of the whole-body SUV distribution outperformed standard uptake metrics (SUV_mean_, SUV_max_) for predicting both early progression and OS, suggesting that intrapatient heterogeneity captures prognostic signal beyond average uptake [15]. Similarly, using fully automated segmentation, Rios-Sanchez et al. found that uptake dispersion, measured as standard deviation of the whole-body SUV distribution, was the strongest univariable predictor of PSA50 response, outperforming volume and mean uptake [16].

The prognostic value of SUV_mean_ may be treatment-dependent. In the ENZA-p trial, where [^177^Lu]Lu-PSMA was combined with enzalutamide as first-line mCRPC treatment, SUV_mean_ had no prognostic or predictive value for OS, PSA-PFS or PSA90. Patients with lower SUV_mean_ even had higher PSA90 response rates in the enzalutamide monotherapy arm, suggesting that patients with lower PSMA expression may benefit from alternative therapies [17]. SUV_mean_ performance may also depend on the radiotracer: in one cohort, it was prognostic for OS with [^68^Ga]Ga-PSMA-11 but not with [^18^F]PSMA-1007 [18].

**Normalized tumor-to-reference ratios**

Plain SUV values can be affected by scanner hardware and calibration, reconstruction and acquisition protocols, which may limit comparability across patients and centers [19,20,21]. Therefore, comparing tumor uptake to a reference organ—the liver, salivary glands or kidneys—may reduce this variability [19]. Physiologic uptake in these reference organs is tracer-dependent—hepatic activity is markedly higher and renal activity is lower for the hepatobiliary-excreted [^18^F]PSMA-1007, so a tumor-to-reference ratio derived with one tracer may not be directly comparable to the same ratio obtained with another. Several studies have tested quantitative tumor-to-reference ratios. Higher tumor-to-salivary-gland scores predicted higher rates of PSA response in two independent cohorts [7,13]. Eisazadeh et al. showed that the hottest-lesion SUL_max_ (standardized uptake value corrected for lean body mass) and the parotid-SUV_mean_ ratio (SUL_max_/parotid-SUV_mean_) were the only PET uptake variables independently associated with OS in multivariable analysis (cutoff 2.4) [22].

The tumor-to-kidney ratio was associated with PFS in one cohort [8] and differentiated PSA50 responders from non-responders in another, where it also correlated with the absorbed tumor dose [23]. Although promising, tumor-to-reference ratios did not outperform conventional SUV metrics in some cohorts [8,13] and their added value over standard uptake parameters remains inconsistent across studies.

**Visual scoring systems**

WB SUV_mean_ measurement is time-consuming and requires dedicated segmentation software that is not universally available. Two visual scoring systems have been proposed as simplified alternatives to capture the WB heterogeneity information on a standard PET/CT workstation.

The visual parotid-salivary-gland score (vPSG) classifies PSMA expression based on parotid uptake on MIP images [7]. The HIT score is a four-category score combining visual heterogeneity and intensity of tumor [9]. Both showed prognostic value comparable to quantitative SUV_mean_ in their derivation cohorts, with substantial inter-reader agreement (κ 0.68 for vPSG and 0.71 for HIT score, with κ > 0.60 generally considered substantial agreement) [7,9]. However, when applied in an independent US multicenter Expanded-Access Program cohort, both scores predicted PSA response, but neither reliably predicted PSA-PFS or OS as was expected from previous studies; quantitative SUV_mean_ remained a more reliable prognosticator [10].

### 2.2. Volumetric and Whole-Body Tumor Burden Biomarkers

Beyond PSMA expression, the overall quantity of PSMA-positive disease may also affect RLT outcomes. Higher tumor volume generally reflects more advanced and biologically aggressive disease and has been associated with poorer outcomes across multiple systemic therapies. It can also lead to a higher volume of irradiated tissue during RLT, resulting in a lower radiation dose to normal organs due to the tumor sink effect [5,24] but also decreased per-lesion absorbed doses [25] and increased hematologic toxicity especially in the presence of bone metastases [26,27]. The evidence generally supports an association between higher baseline tumor volume and worse survival, but the strength and independence of this effect vary across different cohorts and endpoints.

**Global whole-body tumor volume**

Higher baseline PSMA-positive tumor volume (PSMA-TV) is consistently associated with worse OS across different studies [6,28,29]. A pooled analysis confirmed this association (HR 1.37) with low between-study heterogeneity (I^2^ = 17.1%) [11]. However, in the VISION secondary analysis, PSMA-TV did not remain in final multivariable models—the composite metric tumor load (PSMA-TV × SUV_mean_) was more robust, and regional TV in the liver and soft tissue carried additional independent prognostic value for worse rPFS and OS [6]. In other independent multi-institutional cohorts, composite metrics similarly outperformed pure volume in multivariable models [28], while WB SUV_mean_ consistently remained the strongest independent prognosticator [6,10].

The prognostic value of volumetric metrics appears to depend on the clinical endpoint. Across several cohorts, tumor volume independently predicted OS but not PSA response or PSA-PFS, whereas uptake-based parameters showed the opposite pattern [29,30,31]. This pattern was not universal and may vary across patient populations and study designs. In one small cohort, volumetric metrics were associated with PSA response in univariable analysis [32]. However, the overall pattern supports TV as mainly a prognostic biomarker reflecting the disease extent, while uptake-based metrics may better reflect treatment-specific PSMA receptor targeting [5].

The ENZA-p substudy suggests that TV may also have predictive value in combination therapy [17]. Patients with higher total TV (TTV) benefited from adding [^177^Lu]Lu-PSMA to enzalutamide (OS 28 vs. 20 months, interaction *p* = 0.0078), whereas patients with lower TTV did not. SUV_mean_ had no prognostic or predictive value in this combination therapy, suggesting that the importance of volume versus uptake biomarkers may be treatment-dependent.

**Burden-derived composite metrics**

One limitation of pure tumor volume is that it represents disease extent but ignores PSMA expression intensity. To address this limitation, several investigators explored composite metrics that integrate both volume and uptake.

Tumor load or total lesion PSMA (TL-PSMA = PSMA-TV × SUV_mean_) combines disease extent with PSMA expression intensity, with higher values reflecting either more extensive disease, higher PSMA expression, or both [28,30]. Total lesion quotient (TLQ = PSMA-TV/SUV_mean_) is a ratio of total tumor volume to average PSMA uptake intensity, where higher values reflect extensive disease with relatively low PSMA expression, an unfavorable combination for RLT [28]. Terminology varies across studies, for example TL-PSMA, TLP, and TLU—all describe the same calculation.

Composite metrics generally outperformed pure tumor volume in several cohorts: TLQ [28,33] or TL-PSMA [30] remained significant independent predictors of OS in multivariable models, while PSMA-TV did not. In the two studies that compared both composite metrics, TLQ outperformed TL-PSMA [28,33]. One possible explanation is that tumor volume and SUV_mean_ have opposing prognostic directions: higher tumor volume predicts worse survival, whereas higher SUV_mean_ is associated with better outcomes. Multiplying these parameters in TL-PSMA may therefore dilute the prognostic signal, whereas dividing tumor volume by SUV_mean_ in TLQ preserves both effects [28]. Whether composite metrics outperform WB SUV_mean_ remains unresolved: SUV_mean_ was the stronger predictor in some cohorts [6,10] but showed no significant association with OS in others where composite metrics did [30,33]. As with pure volume, neither composite metrics predicted short-term outcomes (PSA response) in larger cohorts [29,30] although univariable associations were reported in smaller cohorts [32,34].

### 2.3. Disease Distribution and Clinical–Biological Modifiers

**Metastatic distribution patterns**

Liver metastases are a negative prognostic factor for patients treated with [^177^Lu]Lu-PSMA RLT. Liver involvement predicted worse OS (multivariable HR 4.06) and lower PSA50 response rates in the largest dedicated analysis [35], as well as showing independent associations with worse outcomes in other cohorts [6,36,37]. However, in multivariable nomograms from the VISION trial that incorporated a broader set of clinical and laboratory parameters, liver metastases were retained for rPFS but not for OS [38].

Extensive bone involvement is associated with worse survival [26,27,37,39] and increased hematological toxicity including dose delays/reductions and need for blood transfusions [26,27,39]. However, in a bone-dominant cohort, interim rather than baseline bone tumor volume independently predicted OS [40]. Metastatic distribution patterns consistently predicted OS across individual cohorts, and a recent meta-analysis confirmed worse OS for visceral (pooled HR 1.65, 8 studies, I^2^ = 0%), liver (HR 2.15, 9 studies, I^2^ = 25.8%), and bone metastases (HR 2.09, 6 studies, I^2^ = 45.6%), with lower between-study heterogeneity than reported for quantitative uptake biomarkers [41].

**Discordant disease**

Beyond anatomical distribution, the biological phenotype of metastatic disease—specifically the presence of FDG-avid and PSMA-low uptake lesions reflecting tumor dedifferentiation and loss of PSMA expression—can independently predict poor RLT outcomes. Among VISION-eligible patients, quantitatively defined discordant disease (any FDG-avid lesion with PSMA uptake SUV_max_ < 10) was associated with worse OS (multivariable HR 3.0, *p* = 0.009) and PSA-PFS (HR 2.4, *p* = 0.008), independent of clinical covariates [42]. Similar findings were reported in cohorts using visual assessment—both visual FDG-dominant disease and visual categorization of any FDG+/PSMA− lesion independently predicted worse OS [43,44]. At the lesion level, the ratio of FDG to PSMA uptake (FPQ), rather than FDG uptake alone, predicted which individual lesions would progress under RLT [45].

**Imaging-based patient selection**

The TheraP trial used dual FDG and PSMA tracer imaging to exclude patients with low PSMA expression (SUV_max_ < 20 at the highest-uptake site or SUV_max_ ≤ 10 at measurable lesions) or discordant FDG+/PSMA− disease, who had significantly shorter survival than enrolled patients (restricted mean survival time 11.0 vs. 18.8 months) [3]. In a real-world cohort, quantitative PSMA uptake thresholds used in the TheraP trial were combined with contrast-enhanced CT to identify PSMA-negative lesions and independently predicted better OS (multivariable HR 0.5) [46]. When the exclusion criteria were analyzed separately (low PSMA uptake vs. FDG+/PSMA− discordance), the worse outcomes were driven by discordant disease, whereas low PSMA uptake alone did not affect OS (HR 1.1, *p* = 0.9) [42].

Even among patients with concordant FDG+/PSMA+ disease, FDG-based volumetric parameters may provide additional prognostic information: higher total FDG metabolic tumor volume (≥200 mL) predicted worse PSA response, rPFS [12] and OS (HR 2.28) regardless of the treatment arm [3]. In a dual-tracer cohort, FDG total tumor volume was the only independent predictor of OS, while PSMA-SUV_mean_ predicted biochemical response but not survival [18]. This is consistent with the pattern observed for PSMA-derived metrics, in which volumetric burden is associated primarily with prognosis and uptake-based parameters with biochemical response.

**Clinical and biological modifiers**

Disease stage, tumor differentiation, overall disease extent, the distribution of visceral, liver and bone metastases, and FDG-avid, PSMA-low discordant disease, together with prior and ongoing systemic therapies, performance status, and laboratory parameters such as hemoglobin, ALP, LDH, PSA and inflammatory markers, may all influence both PSMA expression and outcome after RLT.

Androgen deprivation and androgen-receptor pathway inhibitors may increase or decrease PSMA expression depending on castration status and the duration of treatment [47,48], while taxane chemotherapy may reduce PSMA uptake [49], meaning that prior therapies can affect PET/CT biomarkers. Clinical and laboratory variables are also prognostically important: in the VISION trial they alone predicted overall survival as accurately as models that also included PSMA PET/CT parameters [38]. Therefore PSMA PET/CT biomarkers cannot be interpreted in isolation from this clinical context.

## 3. Methodological Heterogeneity, Clinical Readiness, and Priorities for Standardization

### 3.1. Sources of Methodological Heterogeneity

Although the previously discussed PET/CT biomarkers show broadly consistent prognostic associations with RLT outcomes, the studies reporting them differ in nearly every methodological step of the imaging pipeline. As a result, reported quantitative thresholds are often not transferable between centers or studies [11]. Table 1 illustrates this variability across studies evaluating WB SUV_mean_.

**(a)**  **Radiotracer variability**

The reviewed studies evaluating the predictive and prognostic value of PET/CT biomarkers used different diagnostic radiotracers, which differ in pharmacokinetics, biodistribution, excretion route and recommended uptake times [19]: [^68^Ga]Ga-PSMA-11 (majority), [^18^F]PSMA-1007 [15,36], [^18^F]flotufolastat [29], and several mixed-tracer cohorts (Table 2). Additionally, tracers differ in physiologic background uptake, notably higher hepatobiliary activity with [^18^F]PSMA-1007, which can affect lesion detectability, image interpretation, and the suitability of the liver as a reference organ for patient selection and liver-based segmentation thresholds. One study directly compared prognostic performance across tracers and found that WB SUV_mean_ was associated with OS in patients imaged with [^68^Ga]Ga-PSMA-11 (*n* = 107) but not in those imaged with [^18^F]PSMA-1007 (*n* = 45) [18]. Several studies included multiple PSMA tracers within the same cohort without stratified or sensitivity analyses by tracer type, which may introduce additional measurement variability in uptake-based biomarkers [10,26,42]. Consequently, quantitative SUV thresholds established for one PSMA tracer cannot be assumed to be directly transferable to other tracers without dedicated validation.

**(b)**  **Scanner hardware and reconstruction**

PET/CT scanner technology and image reconstruction algorithms further contribute to this variability. For example, Bayesian penalized likelihood (BPL) reconstruction has been shown to produce systematically higher SUV_max_ values compared with conventional ordered-subset expectation maximization (OSEM). In one study, it was shown that the BPL method produced higher SUV_max_ values by an average of +2.98 in [^68^Ga]Ga-PSMA-11 scans when compared to OSEM although the overall inter-method variation remained below 10% [20]. Such differences may influence uptake-based biomarkers and the thresholds derived from them. In contrast, scanner hardware itself appears to contribute less systematic bias, as no significant difference in SUV repeatability was observed between two different PET/CT scanners [21]. Nevertheless, scanner-specific acquisition and reconstruction settings may still affect quantitative measurements and therefore require transparent reporting.

Despite the known impact of reconstruction on quantitative PET metrics, the majority of reviewed studies did not report PET reconstruction parameters. Among those that did, methods ranged from standard OSEM [42] through OSEM with resolution recovery and time-of-flight corrections [15,29] to BPL [26,51].

Quantitative harmonization frameworks may help reduce variability across scanners and reconstruction protocols. EARL accreditation or equivalent phantom-based harmonization procedures are designed to improve the comparability of SUV measurements between centers. However, only two of the reviewed studies documented harmonization measures such as EARL accreditation [22] or phantom-based harmonization [17]. Notably, none of three largest multicenter studies documented inter-scanner harmonization [6,7,37], and reported the use of different reconstruction methods across participating centers [7,37]. As a result, reconstruction-related variability may exist not only between studies but also within multicenter cohorts.

**(c)**  **Acquisition protocol differences**

Acquisition protocols are a further source of variability. Parameters such as injected activity, uptake time, and scan coverage can influence measured SUV values and whole-body tumor burden estimates.

Among the reviewed studies, protocols for [^68^Ga]Ga-PSMA-11 tracer were broadly comparable. Injected activity was weight-based and average uptake time was approximately 60 min across the studies, in line with procedural guidelines. However, acquisition parameters were not consistently reported across studies, limiting the ability to assess comparability. Scan coverage showed greater variability. While some studies acquired images from the skull base to the proximal femur [22], others used a wider field of view extending from the vertex to the knees [51]. Differences in scan range may influence whole-body volumetric biomarkers, as wider coverage can capture additional metastatic sites such as lesions in the skull or distal extremities. Consequently, whole-body tumor volume measurements derived from studies using different scan coverage may not be directly comparable.

**(d)**  **Segmentation methods and thresholds**

Segmentation methods seem to be the largest source of methodological heterogeneity between the studies, affecting quantitative WB PET/CT imaging biomarkers. The selected segmentation threshold typically refers to the SUV cutoff used to define the tumor lesions—voxels above the threshold are included in the tumor volume. This principle directly affects the WB quantitative metrics such as PSMA-TV, TL-PSMA, TLQ and WB SUV_mean_, as a lower threshold includes more low-uptake voxels, resulting in lower average uptake with increased volume, and vice versa.

Across the reviewed studies, segmentation approaches varied widely (Table 1). At least 17 different software platforms were used with at least 6 different threshold concepts: fixed absolute SUV (e.g., SUV ≥ 3, ≥2.5) [9,15,17,26,36,40,42], fixed relative SUV (a percentage of local SUV_max_, e.g., 40%, 45%) [13,22], hybrid approaches combining two different threshold methods within a single segmentation workflow [6,30,51], liver-based adaptive thresholds [8,10,16,28,34], fully automated deep learning without any predefined threshold [33], and software-internal thresholds not explicitly reported [7,29,37,39].

Each method defines tumor tissue differently and therefore produces different quantitative biomarker values. Fixed absolute thresholds apply the same SUV cutoff across all patients regardless of individual biodistribution. Liver-based adaptive thresholds vary between patients and may be influenced by the tumor sink effect: in patients with high tumor burden, reduced hepatic background uptake lowers the liver-derived threshold, which can lead to overestimation of segmented tumor volume. Relative percentage-of-local-maximum thresholds depend on each lesion’s intensity with potential to underestimate the volume of highly intense lesions while overestimating the volume of low-intensity lesions. Hybrid workflows combine two of these methods for different tissue compartments, introducing further variability, while deep learning and proprietary platform-based segmentations use undisclosed or data-driven boundaries that cannot be directly compared with any of the above.

Even when similar segmentation approaches are used, derived biomarker thresholds may differ substantially between studies. Two investigations used the same software (MIWBAS v1.0) and the same liver-based segmentation method with the same statistical cutoff derivation methods, both evaluating OS in [^68^Ga]Ga-PSMA-11 cohorts with partly overlapping patients, yet derived different PSMA-TV cutoffs of 41.1 mL [28] and 290.6 mL [8] for OS. This illustrates that internally derived thresholds may depend strongly on cohort characteristics and statistical methods, emphasizing the need for external validation before clinical application.

Another frequently underreported segmentation parameter is the minimum lesion size or volume. This threshold determines whether small lesions are included, thus directly affecting all WB quantitative metrics. Among the few studies reporting this parameter, minimum lesion volume ranged from 0.2 mL [17,30] to 0.5 mL [28] while one study specified a minimum lesion size of 0.5 mm [9], yet the majority of studies did not report it at all.

Without taking into account the methodology components such as software platform, segmentation and volume threshold, segmentation-dependent PET/CT biomarkers are difficult to directly compare across studies. The wide range of published cutoffs across outcomes—41.1 to 394.1 mL for PSMA-TV [8,17,28,29,40], 4.1 to 127.2 for TLQ [28,34], and 6.95 to 13.02 for SUV_mean_ [13,34] likely reflects different methodological choices in segmentation and threshold definition rather than different tumor biology. This problem is further complicated by the fact that the majority of reported cutoffs were internally derived using heterogenous post hoc methods and lacked external validation, increasing the risk of overfitting to individual datasets.

**(e)**  **Outcome measures and response criteria**

Heterogeneity also arises from the choice of clinical endpoints used to evaluate imaging biomarker performance. Different endpoints capture distinct aspects of treatment benefit and therefore may yield different conclusions regarding the prognostic or predictive value of a given biomarker.

The reviewed studies evaluated biomarker performance against a wide range of endpoints that measure different clinical outcomes. The most commonly evaluated endpoints were OS and PSA50, but also PSA-PFS [7,9,10,12,17,29,34,37,40,42,43], rPFS [6,38], molecular imaging response (RECIP) [13,15,22,51], PERCIST-based lesion-level progression [45], and hematological toxicity [26,39].

A biomarker that predicts PSA50 may not predict rPFS or OS, thus studies using different outcomes cannot be directly compared. Even within the same study, biomarker performance varied by endpoint: TTV independently predicted OS but not PSA response or PSA-PFS [29], MTV was associated with OS but could not predict PSA response [30], and the tumor-to-kidney uptake ratio was significant for PFS but not for OS [8]. Across studies, the same biomarker evaluated against different endpoint definitions can yield apparently contradictory conclusions—for example, higher SUV_mean_ predicted any PSA decrease at 8 weeks [36], PSA50 at any time during the treatment [9], but not PSA90 [17], yet all three studies evaluated “PSA response” as an endpoint.

Even among studies evaluating the same biomarker for the same endpoint, the covariates included in multivariable models differed substantially. Taking WB SUV_mean_ and OS as an example, the multivariable models differed: Kuo et al. included only the treatment arm with no clinical covariates [6]; Gafita et al. included time since diagnosis, prior chemotherapy, lesion count, liver metastases, bone involvement, nodal metastases and hemoglobin [37]; Hartrampf et al. included age, liver metastases, CRP, hemoglobin, and LDH [36]; and Kimura et al. included age, time since diagnosis, prior systemic therapies, hemoglobin, thrombocytes, and baseline PSA [10]. This alters the adjusted effect size and limits direct comparison of hazard ratios across studies.

Similar variability applies to clinical covariates themselves: bone involvement—consistently identified as a prognostic factor—was defined as binary presence [37], lesion count categories [27], percentage of total bone volume with PSMA-avid disease [26], quantitative bone-compartment metrics [39], or categorical burden classification [51], with no study systematically comparing these approaches.

Finally, molecular imaging response criteria (RECIP) were used in few studies [13,15,22,51]. RECIP criteria are relatively new and not yet widely adopted, but represent an important step toward standardized molecular imaging response assessment in PSMA-targeted therapy [53,54,55]. However, the timing of follow-up imaging varied across these studies, further limiting comparability.

Overall, variability in endpoint definitions, response criteria, and multivariable modeling strategies complicates interpretation of imaging biomarker studies and contributes to the inconsistent effect estimates reported in the literature.

### 3.2. Limitations for Clinical Implementation

The methodological variability outlined above has measurable consequences when results from different studies are compared or pooled. Even when the direction of biomarker associations appears consistent, substantial between-study heterogeneity limits the clinical applicability of reported quantitative thresholds.

In a recent meta-analysis, Kiani et al. pooled SUV_mean_ results from six studies for OS and found striking between-study disagreement (I^2^ = 93.4%), despite a significant result (HR 0.93, 95% CI 0.90–0.96) [11]. In contrast, pooled TV results for OS showed low heterogeneity (I^2^ = 17.1%; HR 1.37, 95% CI 1.27–1.48) [11]. These findings suggest that uptake-based PET biomarkers may be more sensitive to methodological variability than volumetric measures.

Several factors likely contribute to this pattern. Volumetric biomarkers primarily depend on the delineation of tumor boundaries, and regardless of the segmentation method, larger tumor burden consistently reflects more advanced disease and therefore worse survival. WB SUV_mean_, by contrast, is affected by tracer pharmacokinetics, the reconstruction algorithm, segmentation threshold and even the computation method (voxel-weighted vs. per-lesion average), meaning that WB SUV_mean_ reported by different studies is not necessarily the same measurement. Although the direction of the association between higher SUV_mean_ and improved outcomes appears broadly consistent, the magnitude of the effect varies substantially across cohorts.

The studies pooled in the meta-analysis also differed in clinical and treatment context [11]. Within six studies pooled for the WB SUV_mean_ and OS association, therapeutic radioligands included both [^177^Lu]Lu-PSMA-617 and [^177^Lu]Lu-PSMA-I&T, the number of treatment cycles ranged from a median of three [28,36,37] to a median of five with up to 18 cycles administered [30], and study designs ranged from a phase 3 RCT [6] to single-center retrospective analyses [36]. Within patient populations, prior chemotherapy rates varied from 65.7% [30] to 100% [6]. Covariates included in multivariable analysis also differed: Kuo et al. adjusted only for the treatment arm with no clinical covariates [6], Ferdinandus et al. performed no multivariable analysis [50], while Gafita et al. included seven clinical variables [37]. The resulting HR for the same biomarker therefore depends on what covariates are included in the model.

By contrast, pooled estimates for clinical prognostic variables show much lower between-study disagreement: in a meta-analysis of 32 PSMA-RLT studies, prior chemotherapy (I^2^ = 0%), ECOG performance status (I^2^ = 0%), and liver metastases (I^2^ = 25.8%) all produced consistent pooled estimates for OS, while SUV_mean_ showed I^2^ of 79.6% [41], consistent with the Kiani et al. findings and indicating that the between-study imaging biomarker disagreement cannot be explained by differences in patient populations alone.

To date, no quantitative cutoff has been externally validated for clinical implementation. The closest to external validation is the continuous nomogram by Gafita et al. [37], which was developed exclusively in [^68^Ga]Ga-PSMA-11 cohorts (patients imaged with ^18^F-labeled tracers were excluded), and showed moderate discrimination when applied to the prospective [^68^Ga]Ga-PSMA-11-based VISION trial data (OS C-index 0.67) [38] and to a real-world cohort (C-index 0.70) [52]. Gafita et al. cautioned that applying the model to patients imaged with ^18^F-labeled tracers could overestimate prognosis, and no equivalent for ^18^F-labelled tracers has been externally validated. However, this model produces a continuous risk estimate rather than a binary threshold suitable for simple treatment selection. Consequently, patient eligibility for PSMA-RLT in clinical practice continues to rely primarily on visual PSMA PET assessment as defined in the VISION trial [1]. Importantly, the clinical context in which RLT is applied may also influence biomarker associations. In the ENZA-p trial, for example, WB SUV_mean_ did not retain prognostic or predictive value when [^177^Lu]Lu-PSMA was combined with enzalutamide in the first-line mCRPC setting [17]. This observation suggests that biomarker associations derived from RLT monotherapy cohorts may not necessarily translate to combination therapies or earlier disease settings.

### 3.3. Priorities for Standardization: A Path Forward

Addressing the methodological variability outlined above is essential for translating PSMA PET-derived biomarkers from research observations into clinically applicable tools for patient selection. Several methodological aspects should be prioritized in future studies to improve the reproducibility and comparability of results.

**Acquisition and reconstruction**

Reproducible quantitative imaging biomarkers require standardized acquisition and reconstruction; therefore, future studies should follow the EANM/SNMMI procedure guidelines [19], specifying the tracer used, injected activity, uptake time, scan coverage, and reconstruction algorithm. EARL accreditation [19] or equivalent phantom-based harmonization would improve quantitative comparability across scanners and centers—particularly important for multicenter studies, but also beneficial within single-center settings where scanner upgrades or protocol changes occur over time. Given the known tracer-dependent differences in SUV values, quantitative results from different PSMA radiotracers should not be pooled without established cross-tracer comparability; until such data are available, tracer-specific analyses are preferable.

**Segmentation and biomarker definitions**

Segmentation methodology was the largest source of variability identified above, and future studies should therefore specify segmentation workflows, including the software platform used, the thresholding method applied to define tumor tissue, and the minimum lesion size included in the analysis. These parameters directly determine the resulting quantitative biomarker values and are essential for reproducibility. Fully automated segmentation approaches may reduce inter- and intra-operator variability and facilitate large-scale analyses [33]. However, such approaches require multicenter validation and transparent methodological reporting before they can replace conventional threshold-based segmentation methods.

Equally important is reporting biomarker definitions and their calculations: studies reporting the same metric can measure it differently—for example, studies reporting the WB SUV_mean_ biomarker used at least two different calculations: voxel/volume-weighted whole-body average [6,17,37] or per-lesion arithmetic mean [8]. A volume-weighted average gives greater influence to larger lesions because they contribute more voxels, whereas a per-lesion mean gives each lesion equal weight regardless of size, so the same data can produce different values. The same applies to FDG/PSMA discordance, where no consensus exists on what constitutes ‘PSMA-negative’—definitions ranged from quantitative thresholds (e.g., SUV_max_ below a fixed value) [3,42] to visual assessment [43,44] and quantitative lesion-level FDG/PSMA ratios [45], making discordance rates non-comparable across studies. Harmonized terminology is also needed, as identical metrics appear under different names (e.g., TL-PSMA, TLP, and TLU all describe the same calculated biomarker integrating tumor volume and SUV_mean_).

**Clinical context and endpoints**

Imaging biomarkers should be evaluated within a consistent clinical context. Several clinical and laboratory variables were consistently identified as independent prognostic factors across prognostic models and meta-analyses of PSMA-RLT [33,37,38,41,56,57]. To facilitate meaningful comparison between studies, future analyses evaluating PSMA PET biomarkers should report a minimum clinical dataset including ECOG performance status; time since diagnosis; prior systemic therapies including chemotherapy status; liver, bone and nodal metastasis status; complete blood count; PSA; ALP; and LDH. Emerging evidence also supports CRP [56]. Without consistent reporting of these variables, the independent contribution of imaging biomarkers cannot be reliably assessed.

Standardization of clinical endpoints is equally important. Across the reviewed studies, identical endpoint names often referred to different definitions or assessment time points. We therefore recommend reporting OS and assessing biochemical response (PSA) 2–3 weeks after the second cycle as recommended by the EANM/SNMMI therapy guideline [54] using PSA50 as the minimum response threshold. Progression criteria should be explicitly specified, preferably according to PCWG3 [58], which was used in the major RLT trials but left unspecified in many of the reviewed studies. These represent a minimum shared set to enable cross-study comparison; individual studies may evaluate additional or alternative endpoints according to their specific objectives.

Molecular imaging response criteria such as RECIP 1.0 provide standardized molecular imaging response assessment [53,55] alongside conventional endpoints and were applied in several reviewed studies; however, the timing of follow-up imaging varied, and studies using RECIP criteria should specify the assessment time point.

Together, standardized acquisition protocols, transparent segmentation methodology, harmonized biomarker definitions, and consistent clinical endpoint reporting would provide the methodological foundation required for prospective validation of PSMA PET-derived patient selection tools. The reviewed biomarkers capture complementary aspects of disease biology—PSMA expression, total tumor burden, and tumor phenotype—yet substantial methodological heterogeneity currently prevents clinical implementation of reported quantitative thresholds. The prognostic and predictive value of each baseline PSMA PET/CT biomarker category, together with the strength of the pooled evidence, is summarized in Table 3.

## 4. Conclusions

Baseline PSMA PET/CT biomarkers—including uptake-based metrics, volumetric measures, composite metrics, and disease distribution patterns—have demonstrated consistent prognostic associations with outcomes following [^177^Lu]Lu-PSMA RLT, but should be interpreted alongside the treatment history and clinical disease context. However, substantial methodological heterogeneity in segmentation, tracer selection, and biomarker definitions currently prevents clinical implementation of quantitative thresholds. Addressing these barriers through standardized acquisition, harmonized segmentation, and external validation is necessary before these biomarkers can serve as reliable patient selection tools. Until then, clinical evidence supports offering RLT to all patients meeting current eligibility criteria. As these biomarkers mature, their role should be to refine patient selection rather than to restrict access to treatment.

## Figures and Tables

**Table 1:** WB SUV_mean_ as a predictor of RLT outcomes: methodological variability and endpoint-dependent results.

Study, Year (*n*)	Tracer	Scanner/Reconstruction	Segmentation (Software)	SUV_mean_	MVA Covariates	PSA	PFS	OS
Kuo, 2024 [6] (*n* = 826)	^68^Ga-PSMA-11	NR	Hybrid: 41% local max (soft tissue) + DL (bone) (VivoQuant, AntsRNet, MIM)	Voxel- weighted	Tx arm only	✓ PSA50 OR 1.34 (RLT arm) 1.23 (overall)	✓ rPFS HR 0.86 (RLT arm) 0.88 (overall)	✓ HR 0.88 (RLT arm) 0.90 (overall)
Gafita, 2021 [37] (*n* = 270)	^68^Ga-PSMA-11	GE Discovery 710; Siemens Biograph mCT; 64; 16/ PSF-TOF (2i or 3i/21s); OSEM3D (2i/24s); VPFXS ^b^	NR, internal (qPSMA)	Voxel- weighted	LASSO: time since dx, chemo, lesions number, liver, bone, nodes, Hb)	✓ PSA50 OR 2.88	✓ PSA-PFS HR 0.92	✓ HR 0.94
Hotta, 2023 [7] (*n* = 237)	^68^Ga-PSMA-11	GE Discovery 710; Siemens Biograph mCT, 64, 16/ PSF-TOF (2i or 3i/21s); OSEM3D (2i/24s); VPFXS ^b^	NR, internal (qPSMA)	Voxel- weighted	None (UVA only)	✓ PSA50 (≥10; predefined)	✓ PSA-PFS HR 1.7 (<10 vs. ≥10)	✓ HR 1.7 (<10 vs. ≥10)
Buteau, 2022 [12] (*n* = 200)	^68^Ga-PSMA-11	NR (11 centers) ^a^	Fixed: SUV ≥3 (MIM)	Voxel-weighted	Tx arm only	✓ PSA50 OR 12.19 (≥10; predefined) vs. 2.22 (<10)	✓ rPFS + PSA-PFS (≥10 vs. <10)	–
Karimzadeh, 2025 [29] (*n* = 188)	^18^F-flotufolastat	Siemens Biograph mCT Flow, Vision/ OSEM (TrueX 4i/8s)	NR, internal (aPROMISE)	NR	None (UVA only)	✓ PSA50 OR 3.96 (per log_2_-unit)	✓ PSA-PFS HR 0.6 (per log_2_-unit)	✗ HR 0.78 (per log_2_-unit)
Emmett, 2025 [17] (*n* = 160)	^68^Ga-PSMA-11	NR (15 centers) ^a^	Fixed: SUV_max_ > 3 and min lesion vol of 0.2 mL (MIM Encore)	Voxel- weighted	Hb, PSA, LDH, albumin (OS only)	✗ PSA90	✗ PSA-PFS	✗
Telli, 2025 [18] (*n* = 152)	^68^Ga- PSMA-11 and ^18^F- PSMA-1007	Siemens Biograph 128 mCT, 64 VISION 600/ NR	Fixed SUV > 3 for bone lesions, liver-adaptive for other regions (PARS; MICIIS 1.0)	Voxel- weighted	PET parameters only	✓ PSA50 (UVA) OR 2.97	—	✓ ^68^Ga-PSMA-11 ✗ ^18^F-PSMA-1007 (UVA)
Swiha, 2024 [9] (*n* = 139)	^68^Ga-PSMA-11	NR	Fixed: SUV_max_ ≥ 3 and min lesion vol of 0.5 mm (MIM LesionID)	Voxel- weighted	None (UVA only)	✓ PSA50 (quartiles)	✓ PSA-PFS (quartiles)	✗ (quartiles)
Rios-Sanchez, 2025 [16] (*n* = 139)	^68^Ga-PSMA	Philips Vereos, Siemens Biograph Vision 64/OSEM-PSF ^b^	Liver-adaptive (TotalSegmentator v2 organ exclusion)	Voxel- weighted	ML (LASSO, SVM): PET metrics, ALP, PSA, time since dx	✓ PSA50	—	—
Hartrampf, 2023 [36] (*n* = 103)	^18^F-PSMA-1007	NR	Fixed: SUV ≥ 3 (FIJI/ImageJ, Beth Israel plugin))	Voxel- weighted	CRP, Hb, LDH, liver mets, age	✓ Any PSA decrease (8 wk) OR 1.18	—	✓ HR 0.91 (9.4; internally derived)
Hein, 2024 [30] (*n* = 102)	^68^Ga-PSMA-11	Siemens Biograph 40 mCT/OSEM 3i/24s	Hybrid: SUV ≥ 3 + liver-adaptive (1.5 × liver) (Syngo.via)	Voxel- weighted	None (UVA only)	✓ PSA50 (mean: 8.95 vs. 7.9)	—	✗ HR 1.001
Borges, 2025 [26] (*n* = 96)	^68^Ga-PSMA-11 and ^18^F- DCFPyL	GE Discovery MI (4-ring)/ Q-Clear β = 400 ^b^	Fixed: SUV > 3 (aPROMISE + manual)	NR	None (UVA only)	✓ PSA50 (median 8.7 vs. 6.5)	NR	NR
Kimura, 2026 [10] (*n* = 88)	^68^Ga-PSMA-11 and ^18^F-DCFPyL	NR	Liver-adaptive (Hermes Affinity)	Voxel- weighted	Age, time since dx, prior Tx, Hb, platelets, PSA)	✓ PSA50 (median: 11.5 vs. 7.0)	✓ PSA-PFS HR 0.58 (per IQR; 7.8 internally derived)	✓ HR 0.54 (per IQR)
Nikoukar, 2025 [8] (*n* = 82)	^68^Ga- PSMA-11	Siemens Biograph mCT 128/ NR ^b^	Liver-adaptive + 50% local max (MIWBAS v1.0)	Per-lesion arithmetic mean	None (UVA only)	—	✓ PFS (biochem. + imaging) (>10.7; internally derived)	✓ (>10.7; internally derived)
Demirci, 2025 [42] (*n* = 75)	^68^Ga-PSMA-11 and ^18^F-DCFPyL	GE Discovery MI (5-ring)/ OSEM 2i/34s; Sharp IR	Fixed: SUV > 3 (MIM)	NR	Discordance, liver, TTV, LDH, Hb, ECOG, chemo lines, time since dx ^c^	— ^c^	✓ PSA-PFS ^c^ HR 0.80	✗ ^c^ HR 0.88
Koh, 2025 [34] (*n* = 71)	^68^Ga-PSMA-11	NR	Liver-adaptive (Volumetrix MI)	Voxel- weighted	None (UVA only)	✓ PSA50 OR 1.34 (≥6.95; internally derived)	✓ PSA-PFS HR 0.91 (≥8.4; internally derived)	✓ HR 0.49 (>10.2; internally derived)
Kind, 2025 [15] (*n* = 59)	^18^F-PSMA-1007	Philips VEREOS Digital/ BLOB-OS-TF 3i/9s ^b^	Fixed: SUV ≥ 2.5 (MITK Workbench)	Voxel- weighted	Age, prior taxane	✓ Early PD (biochem. + imaging) OR 0.67	—	✓ HR 0.82
Kartal, 2026 [40] (*n* = 56)	^68^Ga-PSMA-11	GE Discovery IQ/ Q.Clear β = 350 ^b^	Fixed: SUV_max_ ≥ 3 (LIFEx)	Voxel- weighted	PET parameters only	—	✓ PSA-PFS (UVA); ✗ (MVA) (≤7.65; internally derived)	✗
Ferdinandus, 2020 [50] (*n* = 50)	^68^Ga-PSMA-11	NR	Fixed: SUV > 3 (MIM)	NR	None (UVA only)	—	—	✓ HR 0.89 (10.55; internally derived)
Ozkan, 2025 [13] (*n* = 41)	^68^Ga-PSMA-11	GE Discovery 710/ NR ^b^	Relative: 40% local SUV_max_ (AW Vol. Share 7)	NR	None (UVA only)	✓ PSA50 (≥13.02; internally derived)	—	—
Siripongsatian, 2024 [32] (*n* = 21)	^18^F-PSMA-1007	Siemens Biograph Vision 64/ TrueX + TOF 2i/5s	Aorta-adaptive + 40% local max (Syngo.via)	Voxel-weighted	None (UVA only)	✗ PSA50 (median: 9.4 vs. 13.0)	—	—

^a^ Formal scanner harmonization/certification documented. Guideline-based standardization (EANM/SNMMI) also reported by Gafita (2021) [37] and Hotta (2023) [7]. ^b^ Scan coverage reported: vertex to mid-thigh—Gafita, Kartal, Ozkan; mid-thigh to skull—Hotta, Kind; vertex to knees—Borges, Rios-Sanchez; vertex to proximal lower leg—Nikoukar. ^c^ SUV_mean_ included as covariate, not primary biomarker (primary: FDG+/PSMA-low discordance). Notation: ✓ = significant; ✗ = not significant; — = not evaluated. Effect sizes reported per unit increase (continuous variable) unless otherwise specified. Abbreviations: ALP, alkaline phosphatase; BPL, Bayesian penalized likelihood; CRP, C-reactive protein; DL, deep learning; ECOG, Eastern Cooperative Oncology Group; HR, hazard ratio; IQR, interquartile range; KM, Kaplan–Meier; LASSO, least absolute shrinkage and selection operator; ML, machine learning; MVA, multivariable analysis; NR, not reported; OR, odds ratio; OSEM, ordered-subset expectation maximization; OS, overall survival; PD, progressive disease; PFS, progression-free survival; PSF-TOF, point spread function–time of flight; rPFS, radiographic PFS; RLT, radioligand therapy; SVM, support vector machine; TTV, total tumor volume; Tx, treatment; UVA, univariable; VPFXS, VUE Point FX-S.

**Table 2:** Diagnostic PSMA radiotracers across the reviewed studies.

Diagnostic PSMA Tracer	Studies (*n*)	Stratified/Sensitivity Analysis by Tracer	External Validation
[^68^Ga]Ga-PSMA-11	Kuo 2024 [6] (*n* = 826) Herrmann 2024 [38] (*n* = 551) Gafita 2021 [37] (*n* = 270) Hotta 2023 [7] (*n* = 237) Buteau 2022 [12]/Hofman 2024 [3] (*n* = 200) Emmett 2025 [17] (*n* = 160) Swiha 2024 [9] (*n* = 139) Rios-Sanchez 2025 [16] (*n* = 139) Seifert 2021 [28] (*n* = 110) Karimzadeh 2023 [46] (*n* = 107) Zang 2025 [33] (*n* = 107) Hein 2024 [30] (*n* = 102) Nikoukar 2025 [8] (*n* = 82) Koh 2025 [34] (*n* = 71) Widjaja 2021 [31] (*n* = 71) Cayirli 2025 [51] (*n* = 48) Telli 2023 [43] (*n* = 52) Ferdinandus 2020 [50] (*n* = 50) Kartal 2026 [40] (*n* = 56) Eisazadeh 2024 [22] (*n* = 60) Ozkan 2025 [13] (*n* = 41)	N/A (single tracer)	Yes—Gafita 2021 continuous nomogram, externally validated in VISION by Herrmann 2024
[^18^F]PSMA-1007	Hartrampf 2023 [36] (*n* = 103) Kind 2025 [15] (*n* = 59) Siripongsatian 2024 [32] (*n* = 21)	N/A (single tracer)	No
[^18^F]flotufolastat	Karimzadeh 2025 [29] (*n* = 188)	N/A (single tracer)	No
Mixed: [^68^Ga]Ga-PSMA-11 + [^18^F]PSMA-1007	Telli 2025 [18] (*n* = 152) Michalski 2021 [44] (*n* = 54)	Yes—Telli 2025 only: SUVmean prognostic for OS with (^68^Ga)Ga-PSMA-11 (*n* = 107) but not (^18^F)PSMA-1007 (*n* = 45)	No
Mixed: [^68^Ga]Ga-PSMA-11 + [^18^F]DCFPyL	Borges 2025 [26] (*n* = 96) Kimura 2026 [10] (*n* = 88) Demirci 2025 [42] (*n* = 75) Muniz 2024 [35] (*n* = 37)	No	No
Mixed: [^68^Ga]Ga-PSMA-11 + [^18^F]JK-PSMA-7	Hohberg 2023 [23] (*n* = 30)	No	No
Tracer not reported	Ahmadzadehfar 2021 [27] (*n* = 319) Ghodsi 2026 [52] (*n* = 168)	N/A	No

Abbreviations: *n*, cohort size; NR, not reported; OS, overall survival; PSMA, prostate-specific membrane antigen; SUVmean, mean standardized uptake value.

**Table 3:** Summary of baseline PSMA PET/CT biomarker categories and their clinical implications for [^177^Lu]Lu-PSMA radioligand therapy.

Biomarker Category	Prognostic Value	Predictive Value	Pooled Evidence (HR; I^2^) *
Uptake (SUVmean)	Higher uptake → better OS; OS, PFS and PSA response; direction consistent	PSA response only (TheraP, SUVmean ≥ 10)	OS HR 0.93; I^2^ 93.4% (very high heterogeneity)
Volume (PSMA-TV)	Higher volume → worse OS; OS direction consistent; PFS associations inconsistent	OS benefit from adding RLT to enzalutamide in high-volume disease (ENZA-p)	OS HR 1.37; I^2^ 17.1% (low heterogeneity)
Distribution (visceral/liver/bone)	Worse OS: liver > bone > visceral; low–moderate heterogeneity	Not established	Visceral OS HR 1.65; I^2^ 0%; liver OS HR 2.15; I^2^ 25.8%; bone OS HR 2.09; I^2^ 45.6%
Composite (TL-PSMA, TLQ)	Higher → worse OS; direction consistent	Not established	TL-PSMA OS HR 1.04; I^2^ 32.8%
Tumor-to-reference ratios	Inconsistent	Not established	No pooled estimate
Discordant disease (FDG+/PSMA−)	Worse OS and PSA-PFS; direction consistent	Not established; used for patient selection (TheraP exclusion)	No pooled estimate

* Pooled hazard ratios and I^2^ values are from the meta-analyses by Kiani et al. (uptake, volume, composite) and Yanagisawa et al. (disease distribution); categories without a pooled estimate were reported only in individual cohorts. Abbreviations: CT, computed tomography; FDG, fluorodeoxyglucose; HR, hazard ratio; I^2^, between-study heterogeneity; OS, overall survival; PET, positron emission tomography; PFS, progression-free survival; PSA, prostate-specific antigen; PSA-PFS, prostate-specific antigen progression-free survival; PSMA, prostate-specific membrane antigen; PSMA-TV, PSMA-positive tumor volume; RLT, radioligand therapy; SUV, standardized uptake value; TL-PSMA, total lesion PSMA; TLQ, total lesion quotient.

## Data Availability

No new data were created or analyzed in this study. Data sharing is not applicable to this article.
